# Contribution of CaMKIV to injury and fear- induced ultrasonic vocalizations in adult mice

**DOI:** 10.1186/1744-8069-1-10

**Published:** 2005-03-22

**Authors:** Shanelle W Ko, Talal Chatila, Min Zhuo

**Affiliations:** 1 Department of Physiology, University of Toronto, Centre for the Study of Pain, Faculty of Medicine, Medical Science Building, Room #3342, 1 King's College Circle, Toronto, Ontario, M5S 1A8, Canada; 2 Department of Pediatrics, The David Geffen School of Medicine at UCLA, 10833 Le Conte Avenue, Los Angeles CA 90095-1752, USA

**Keywords:** Ultrasonic vocalization, fear, memory, pain, CaMKIV, mice

## Abstract

Calcium-calmodulin dependent protein kinase IV (CaMKIV) is a protein kinase that activates the transcription factor CREB. Our previous work demonstrated that mice lacking CaMKIV had a defect in fear memory while behavioral responses to noxious stimuli were unchanged. Here, we measured ultrasonic vocalizations (USVs) before and after fear conditioning and in response to a noxious injection of capsaicin to measure behavioral responses to emotional stimuli. Consistent with previous findings, behavioral nociceptive responses to capsaicin were undistinguishable between wild-type and CaMKIV^-/- ^mice. Wild-type animals showed a selective increase in 50 kHz USVs in response to capsaicin while such an increase was absent in CaMKIV^-/- ^mice. The foot shock given during fear conditioning caused an increase in 30 kHz USVs in both wild-type and CaMKIV^-/- ^mice. When returned to the context one hour later, USVs from the wild-type were significantly decreased. Additionally, the onset of a tone, which had previously been paired with the foot shock, caused a significant decrease in USVs during auditory conditioning. CaMKIV^-/- ^mice showed significantly less reduction in USVs when placed in the same context three days after receiving the shock, consistent with the decrease in freezing reported previously. Our results provide a new approach for investigating the molecular mechanism for emotional vocalization in mice and suggest that CaMKIV dependent signaling pathways play an important role in the emotional response to pain and fear.

## Introduction

The transcription factor CREB (cAMP response-element binding protein) has been implicated in several aspects of higher brain function, such as spatial memory, fear conditioning, conditioned taste aversion and social transmission of food preference [1-7]. Recently, Barrot et al. (2002) reported that changes in CREB expression in the nucleus accumbens shell altered behavioral responses to emotional stimuli. Both the rewarding stimulus of morphine and the aversive stimulus of stress activated CRE-mediated transcription in the nucleus accumbens shell [8]. This study suggests an important role for CREB activation in the behavioral responses to emotional stimuli.

CaMKIV, along with other kinases, activates CREB by phosphorylating it at Ser 133. Phosphorylated CREB recruits the transcriptional co-activator CBP (CREB binding protein) which leads to the activation of CRE (cAMP response element) containing promoters and ultimately to gene expression [9-11]. CaMKIV is a calcium dependent protein kinase that is detected predominantly in the nuclei of neurons [12,13]. The nuclear location of CaMKIV, its broad distribution in forebrain areas related to pain, attention and emotion, [10,14-18] and its activation of CREB make it an attractive candidate for the processing of higher brain functions. Our recent study of CaMKIV knockout mice (CaMKIV^-/-^) showed that fear memory was impaired [10,19], while behavioral responses to noxious stimuli were indistinguishable from that of wild-type mice. CREB activation by fear conditioning was absent in the amygdala of CaMKIV^-/- ^mice [10]. This suggests that CREB activation in response to emotional events may be impaired in CaMKIV^-/- ^mice as well. Therefore, we hypothesize that CaMKIV^-/- ^mice may exhibit a defect in behavioral responses to emotional stimuli.

Currently, responses to physiologically important stimuli, such as pain and fear, are based on the assessment of motor activities, such as licking and freezing. Here we propose to look at an additional measure, ultrasonic vocalizations (USVs), in order to uncover emotional responses less related to the motor response. USVs are an important form of communication in rodents [20-22]. USVs from rats have been recorded in response to painful or startling stimuli [23], drugs of abuse [24-26], stressful situations [27], and as a measure of fear memory [28]. USVs are considerably less studied in adult mice. Sonographic analysis of USVs emitted during male-female pairings show that the majority of USVs were at 70 kHz with some calls at 40 kHz [29]. USVs have not been studied in adult mice in response to the noxious stimulus of capsaicin or as a measure of fear memory.

In this study, we measured USVs emitted from isolated, adult male mice in parallel with conventional behavioral parameters to evaluate responses to emotionally relevant stimuli. We report here that USVs emitted from adult male mice may be used as an index of internal emotional states and that CaMKIV has a significant contribution to the USVs produced in response to capsaicin and fear conditioning.

## Experimental procedures

### Animals

CaMKIV^-/- ^mice were derived as described [10] and bred for several generations (F12-F16) on C57Bl/6 background. Control wild-type mice were adult male (8–12 weeks old) C57Bl/6 mice from Jackson Laboratories. All efforts were made to minimize the number of animals used in this study and to minimize their suffering. At the conclusion of experiments, animals were sacrificed by an overdose of inhaled anesthesia (halothane). The animals were housed on a 12 h: 12 h light: dark cycle with food and water available *ad libitum*. All mouse protocols are in accordance with NIH guidelines and approved by the Animal Care and Use Committee of University of Toronto. No visual difference between wild-type and CaMKIV^-/- ^mice is noticeable, and experiments were performed blind to the genotype.

### Open field activity monitor

To record horizontal locomotor activity we used the Activity Monitor system from Med Associates (43.2 × 43.2 × 30.5 cm; MED-associates, St. Albans, VT). Briefly, this system uses paired sets of photo beams to detect movement in the open field and movement is recorded as beam breaks. The open field is placed inside an isolation chamber with dim illumination and a fan. Each subject was placed in the center of the open field and activity was measured for 30, 60 or 120 minutes.

### Ultrasonic vocalizations

Ultrasonic vocalizations (USVs) were detected using four mini-3 ultrasonic detectors (Ultrasound Advice, London UK), each tuned to a different frequency level ranging from 20 kHz to 130 kHz. The detectors were suspended in one row of four along one side of the open field 19 cm from the floor. The total duration and number of USVs were measured using UltraVox data acquisition software (Noldus Information Technology, Leesburg, Va.). Each transition from vocalization to silence is time stamped, written as a new record and exported into a database for analysis. Before each experiment, the ultrasonic detectors were calibrated so that no USVs were recorded when no animal was present in the chamber. Four frequencies were recorded for each experiment. We set our recording frequencies to represent the range of sound possible from mice. The ultrasonic detectors detect signals within 4 kHz of the set dial frequency. We therefore represent a range from 26 to 124 kHz with our frequency settings at 30, 50, 90, and 120 kHz. The individual frequencies were selected both to reflect a wide distribution of calls and to mirror work established in rats which report USVs emitted selectively at 22 and 50 kHz [25,30]. We choose to focus on USVs at 30 kHz since preliminary studies showed only a small amount of USVs detected at 20 kHz. USV data are presented as a rate (ms/sec).

### Fear conditioning

All mice were habituated to the test chamber for 3 minutes before conditioning. During conditioning, mice were presented with a 30 s tone, the conditioned stimulus (85 dB, 2800 Hz) and 28 s later received the foot shock (0.75 mA) for 2 seconds, unconditioned stimulus. Animals were returned to the same chamber 1 hour, 1, 3, and 7 days later and USVs were recorded. One hour after being placed in the contextual environment, animals were placed into a novel chamber for a total of 6 min and the tone was played for the last 3 minutes. USVs were recorded in the novel environment before and during the tone 1 hour, 1, 3, and 7 days after training.

### Treatments

All animals were gently restrained by the experimenter before intraplantar injection. Capsaicin (1 μg/10 μl) was dissolved in 7.5 % DMSO and injected into the right hind paw at a volume of 10 μl. Formalin (5%, 10 μl) was dissolved in saline and injected into the right hind paw at a volume of 10 μl. Saline was injected as a control.

### Data analysis and statistics

Open field activity was analyzed using the Activity monitor program. USVs were analyzed using the UltraVox program. Statistical comparisons were made using the t- test, paired t-test or One way or two way ANOVA (Student-Newmann-Keuls test was used for *post hoc *comparison). All data is represented by the mean +/- S.E.M. In all cases, p < 0.05 is considered statistically significant.

## Results

### USVs from adult male mice in a novel, open field

**Figure 1:**
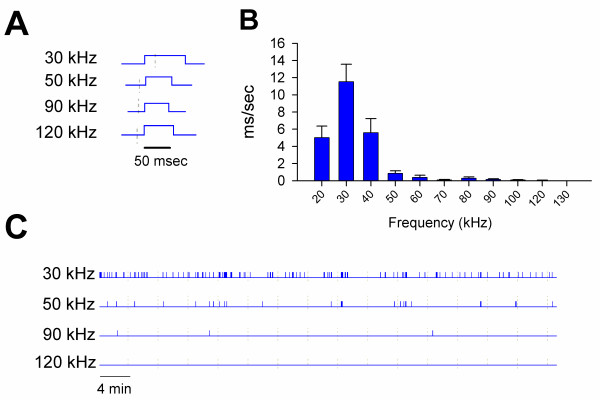
**USVs from adult male mice isolated in an open field**. **A**, Examples of single USVs detected at 30, 50, 90, and 120 kHz. **B**, USVs detected at frequencies ranging from 20 to 130 kHz **C**, Representative trace of USVs at 30, 50, 90, and 120 Hz. Each vertical mark represents a USV at the indicated frequency, with the width representing call duration. Error bars represent S.E.M

To measure USVs from an isolated adult mouse, we placed the animal in an open field arena contained within an isolation chamber. The open field is frequently used to measure locomotor activity as well as anxiety. USVs were detected using four mini-3 ultrasonic detectors, each tuned to a different frequency. Each detector is capable of measuring USVs within 4 kHz of the set dial frequency. Therefore, 30 kHz USVs represent a range between 26–34 kHz USVs, but we will present USV data at the set dial frequency. Since USVs have not been widely reported in isolated adult mice, we first measured ultrasonic emissions at a range of frequencies (from 20 to 130 kHz, Figure 1B). These frequency settings were chosen to cover a wide range and to mirror work done in rats, which suggest the selective modulation of USVs at 22 and 50 kHz.

During 30 minutes in the open field, mice emitted significantly more USVs at 30 kHz (n = 17) compared to 20 (n = 8) or 40 kHz (n = 9) (p < 0.05 for both, Figure 1B). In general there were very few USVs at frequencies higher than 50 kHz. For example, the highest rate of USV production was at 30 kHz (mean 11.52 ± 2.04 ms/sec, n = 17), which was significantly higher than USVs at 50 and 90 kHz (mean 0.53 ± 0.19 ms/sec; 0.14 ± 0.09 ms/sec, respectively, n = 9 and 7, p < 0.001) (Figure 1B–C). Only one 120 kHz USVs was detected (mean 0.03 ± 0.03 ms/sec, n = 7). There was also a significant difference in the duration of a single USV between frequencies. USVs at 30 kHz (83 ± 9 ms) were significantly longer than USVs at 50 kHz (56 ± 2 ms), 90 kHz (49 ± 4 ms), and 120 kHz (60 ± 9 ms) (Figure 1A).

### USVs induced by a noxious stimulus

**Figure 2:**
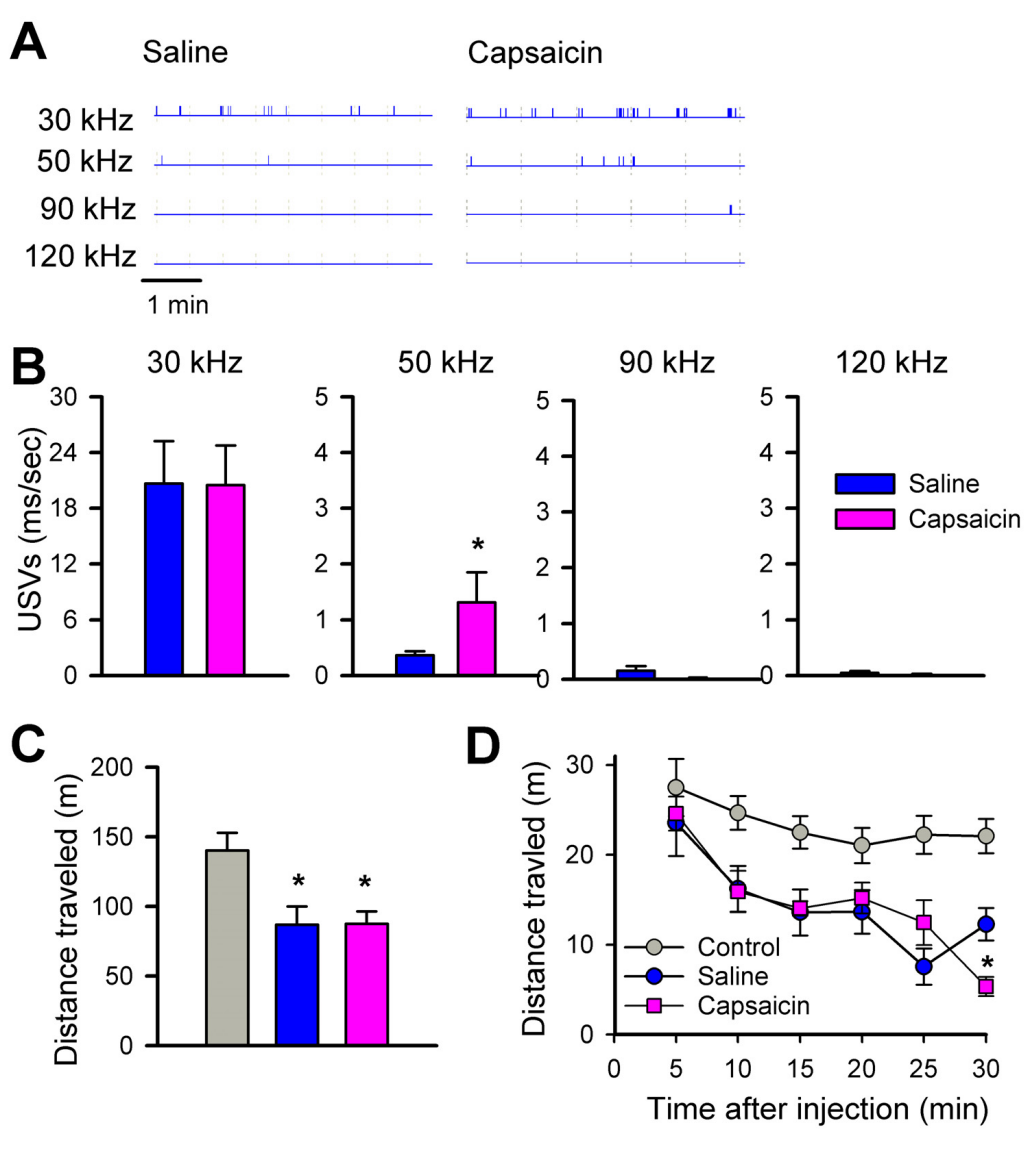
**Subcutaneous injection of capsaicin increased USVs selectively at 50 kHz**. **A**, Representative trace of USVs at 30, 50, 90, and 120 Hz for saline and capsaicin injected mice. **B**, USVs at each frequency for mice injected with saline (hatched bars, n = 19) or capsaicin (black bars, n = 16); capsaicin significantly increased USVs at 50 kHz over saline injected mice (p < 0.001). **C**, Distance traveled in control (white bar), saline (hatched bar) and capsaicin-injected mice (black bar) for 30 min, significant reduction in locomotion were observed with saline and capsaicin (p < 0.01). **D**, Distance traveled presented in five minute blocks over 30 min. Activity is significantly reduced in capsaicin injected mice during the last five minutes when compared to saline and control mice (p < 0.001). Error bars represent S.E.M.

Since USVs are emitted by rodents in response to a number of negative emotional stimuli [31], we wished to determine if an injection of capsaicin, which selectively activates nociceptive C-fibers [32,33], would alter the rate and frequency of USV production. Previous studies in rats demonstrate that USVs can be selectively modulated by either positive or negative stimuli. In rats, USVs at 22 kHz are thought to index a negative affective state while 50 kHz USVs are thought to be more positive [34,35]. Capsaicin (1 μg/10 μl, n = 7–16) or saline (10 μl, n = 9–19) was injected subcutaneously into the right hind paw and mice were immediately transferred to the testing chamber for 30 min. USVs were similar at all frequencies between saline-injected mice and control mice that did not receive an injection. However, injection of capsaicin significantly increased production of 50 kHz USVs [F(2,20) = 4.02, p < 0.001], while USVs at other frequencies remained unchanged (Figure 2A,B). The increase of 50 kHz USVs was mainly due to an increase in the number of USVs since the duration of a single USV at 50 kHz was not significantly affected by capsaicin (before 56 ± 2 ms vs. after 61 ± 3 ms, n = 6 mice). To determine if the capsaicin induced increase in USVs was selective for 50 kHz, we measured USVs at 40 (n = 7–9) and 60 (7–9) kHz and found no significant difference when compared to saline injected mice (data not shown). USVs and the total distance traveled in the open field were measured simultaneously. Capsaicin and saline-injected mice both showed a significant reduction in locomotion as compared to control mice without any injection [F(2,20) = 7.30, p < 0.01] (Figure 2C,D). During the last 5 min of the 30 min session, capsaicin injected mice traveled significantly less than both saline and control mice [F(2,20) = 21.71, p = 0.001] (Figure 2D).

**Figure 3:**
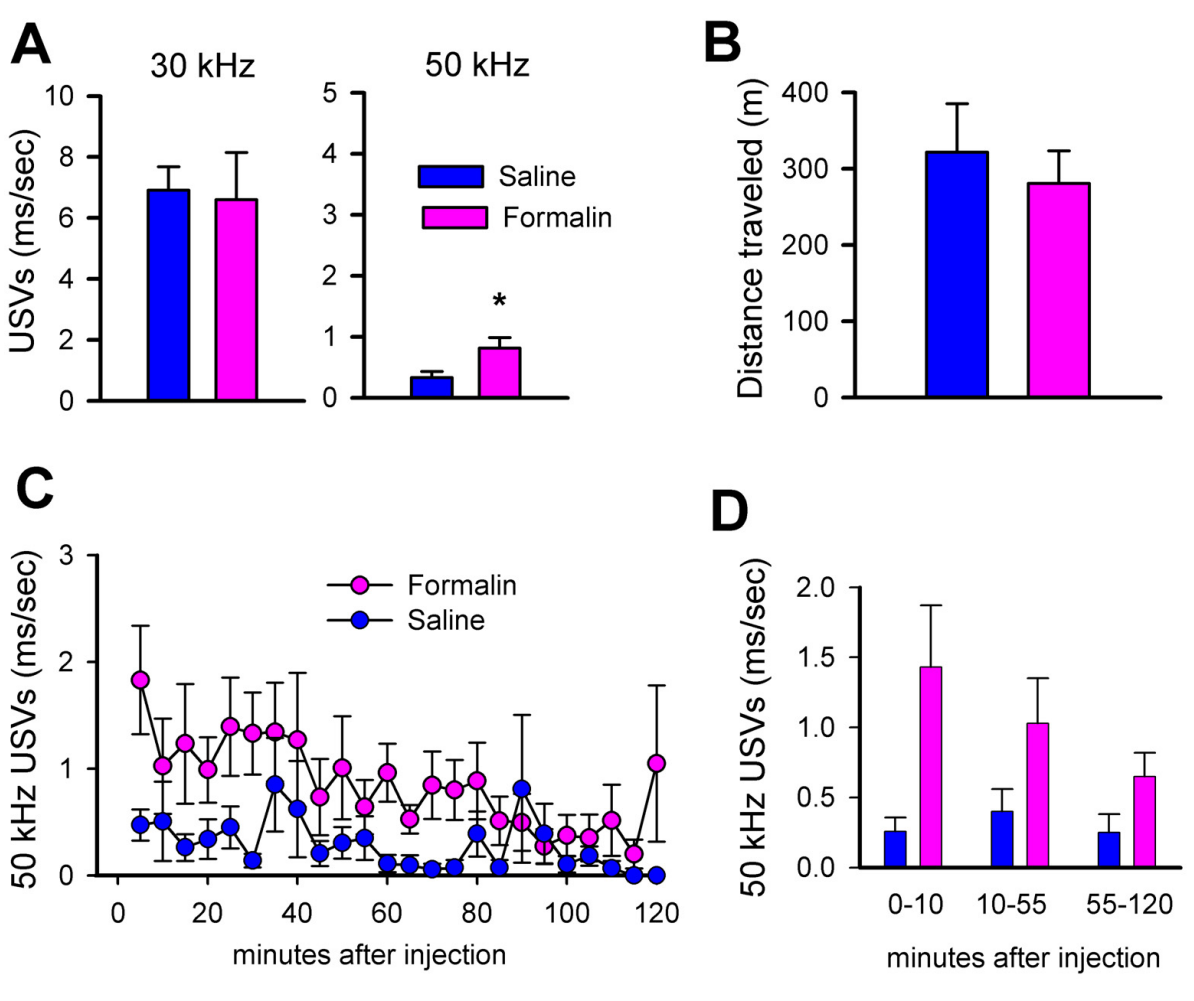
**Subcutaneous injection of formalin results in an increase in 50 kHz USVs**. **A**, While 30 kHz USVs were unchanged, 50 kHz USVs were increased after formalin injection (n = 12 for saline, n = 9 for formalin, p < 0.05) **B**, Locomotor activity was similar between saline (n = 6) and formalin (n = 6) injected mice **C**, 50 kHz USVs presented in five minute blocks over the 120 minute test period. **D**, 50 kHz USVs summarized into three phases of the formalin licking response

To determine if other models of chemical pain could induce 50 kHz USVs, we injected mice with formalin as described [10] and measured USVs for 2 hours. As before, 30 kHz USVs and locomotor activity were similar between formalin (n = 6–9) and saline (n = 6–12) injected animals (Figure 3A,B). Consistent with the capsaicin results, 50 kHz USVs were significantly increased during the two hours after formalin injection (Figure 3A,D). Also, the change in 50 kHZ USVs was selective, since 40 and 60 kHz USVs were similar between saline and formalin injected mice. When the 50 kHz USVs were summarized into the three phases associated with the formalin response [36], a significant effect of the formalin injection was seen across the three phases when compared to saline injected mice (Figure 3D).

### USVs after classic fear conditioning

**Figure 4:**
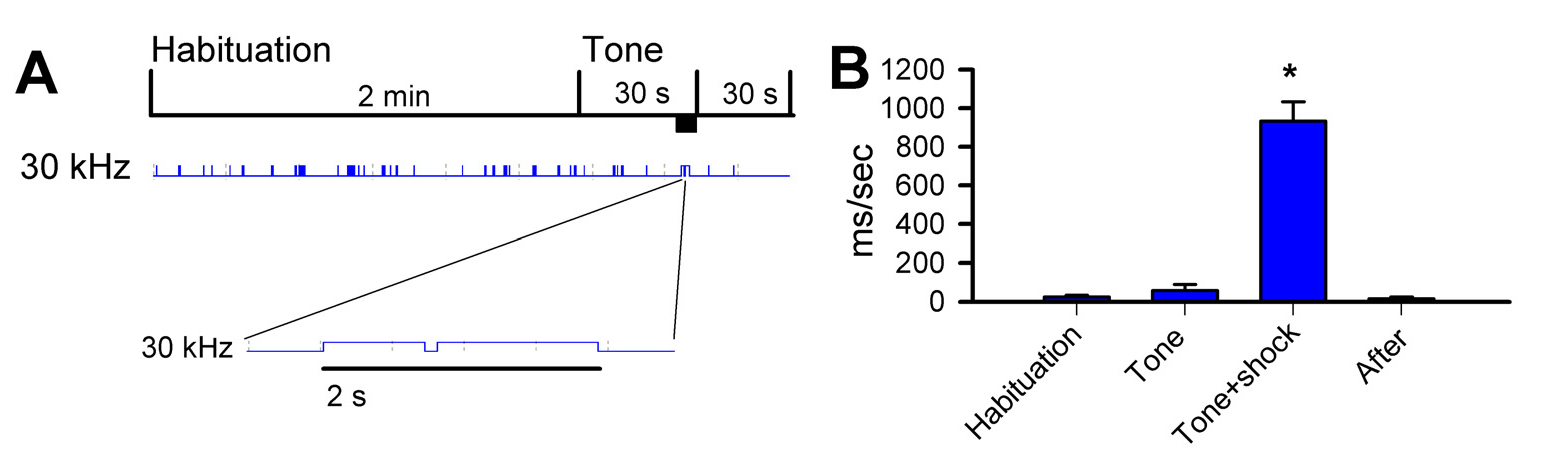
**A single, noxious foot shock increased USVs**. **A**, Top: timeline for the fear conditioning training session. The thick black bar represents the occurrence of the footshock (2 s). Middle: representative USV trace. Bottom: Enlargement of USV trace during the shock period to illustrate the enhanced USV **B**, 30 kHz USVs during the 4 periods of the training session: before, during and after the tone and during the shock. Error bars represent S.E.M.

We measured USVs emitted before and after fear conditioning to determine their role in emotional fear memory. In this behavioral assay, animals learn to fear a neutral conditioned stimulus (tone), which has been paired to an aversive or noxious unconditioned stimulus (foot shock), and the context in which the pairing occurred. After training, animals were placed back into the same environment to test for contextual memory. To assess auditory conditioning, mice were placed in a novel chamber for three minutes before the onset of the tone. First, we examined USVs during classic fear conditioning (Figure 4). After a two-minute habituation period, the tone was played for 28 s before the onset of the foot shock for 2 s; USVs were recorded for an additional 30 s to measure immediate responses. In all experiments, the noxious foot shock significantly increased 30 kHz USVs (n = 17) (Figure 3A,B). The average duration of a single call was also increased (963 ± 158 ms compared to 83 ± 9 ms). The conditioned auditory tone was at 2800 Hz and was not recorded by the ultrasonic detectors. To further determine that the USVs recorded during the shock were not an artifact of the foot shock, we recorded USVs from the empty chamber without a mouse and the chamber containing a deeply anesthetized mouse. Electrical stimulation of the grid floor did not in itself cause any USV. We also administered the shock in the center of the open field and in a foam-lined chamber; the increase in USVs at all four frequencies was seen in both cases.

**Figure 5:**
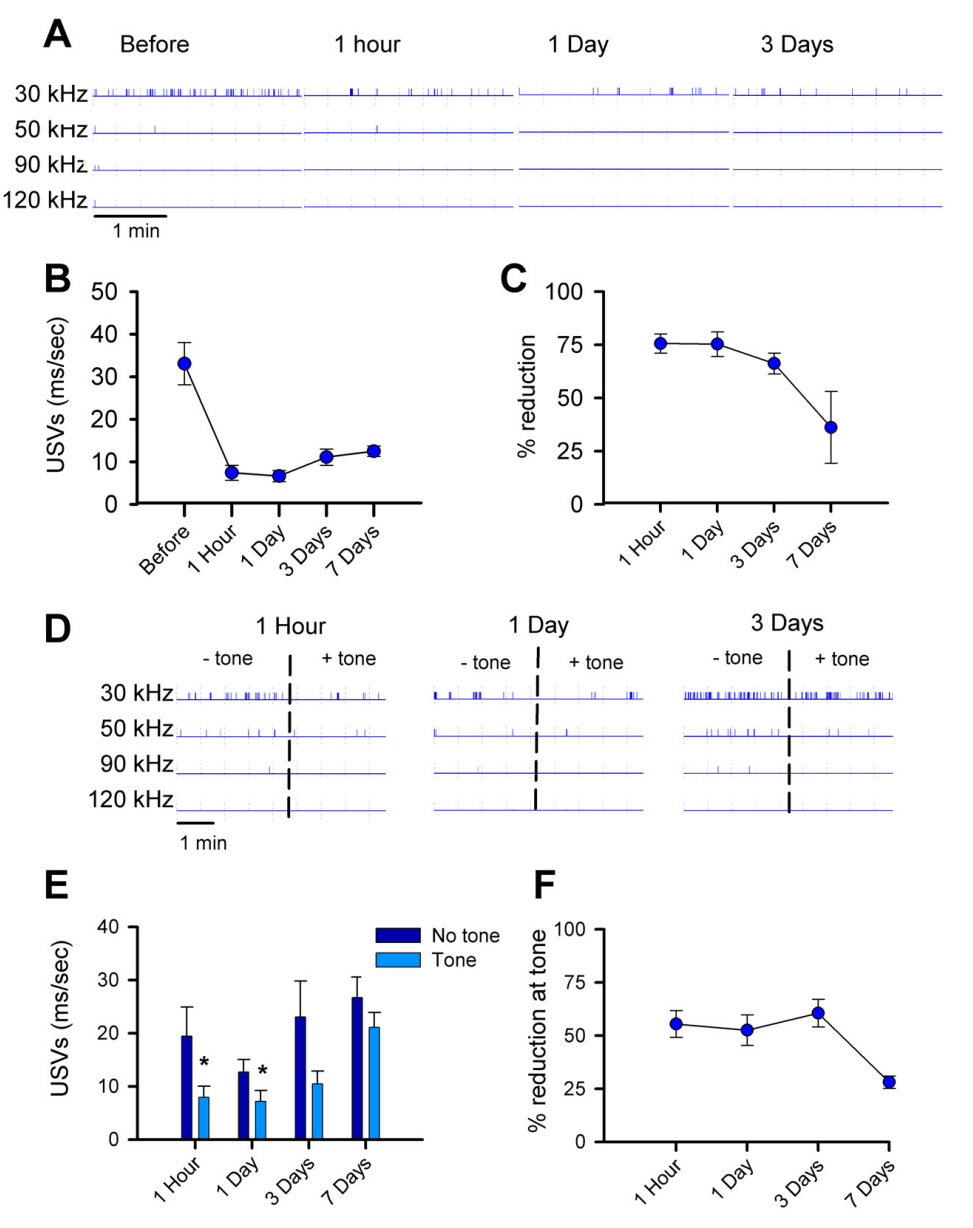
**Reduction in USVs after contextual fear conditioning**. **A**, Representative traces for USVs produced before and 1 h, 1 day and 3 days after training. **B**, 30 kHz USVs during a 3 min exposure to the contextual environment before training and 1 h, 1, 3 (n = 17) and 7 (n = 5) days after. USVs are significantly decreased after training (p < 0.001) **C**, 30 kHz USVs produced in response to the context expressed as the percent reduction from the baseline rate. **D**, Representative traces for USVs produced in a novel environment with and without the tone for 1 h, 1 day and 3 days after training. **E**, 30 kHz USVs for 3 min in a novel context with and without the tone 1 h, 1, 3 (n = 17), and 7 (n = 5) days after training. USVs are significantly decreased at the tone 1 h (p < 0.05) and 1 day (p = 0.01) after training. **F**, 30 kHz USVs produced in response to the tone expressed as a percent reduction from USVs emitted in the novel chamber without the tone. Error bars represent S.E.M

Next, we examined USVs in response to both tone and context at one hour, one, three, and seven days after fear conditioning. Since the vast majority of USVs occurred at 30 kHz, and there was no significant change found at any other frequency, this is the only frequency presented for the fear memory experiments. As shown in Figure 4A and 4B, USVs in the contextual environment were significantly reduced after conditioning when compared to USVs emitted before the training period. USVs were significantly reduced when mice were placed back into the original chamber one hour after fear training and remained at a significantly lower level seven days later [F(4,52) = 21.8, p < 0.001] (Figure 5B). The average reduction in vocalization from baseline recordings was 76 ± 5 % one hour later and decreased to 41.2 ± 13.4 % seven days later (Figure 5C).

In general, USVs were higher in the absence of the tone than when the tone was played. USVs were significantly decreased at the onset of the tone one hour (t = 2.24, p < 0.05), one day (t = 2.75, p < 0.01), but not three and seven days after conditioning (Figure 5D,E). Some animals emitted more USVs in the last three minutes than in the first (4 of 17 mice 1 hour after training). This added high variability when calculating the percentage of reduction at the tone, therefore, these few animals were excluded from Figure 4F. The tone decreased USVs by 55.4 ± 6.3 % one hour after conditioning; seven days later USVs were only decreased by 28.1 ± 2.9% (Figure 5F).

### CaMKIV^-/- ^mice in a novel open field

**Figure 6:**
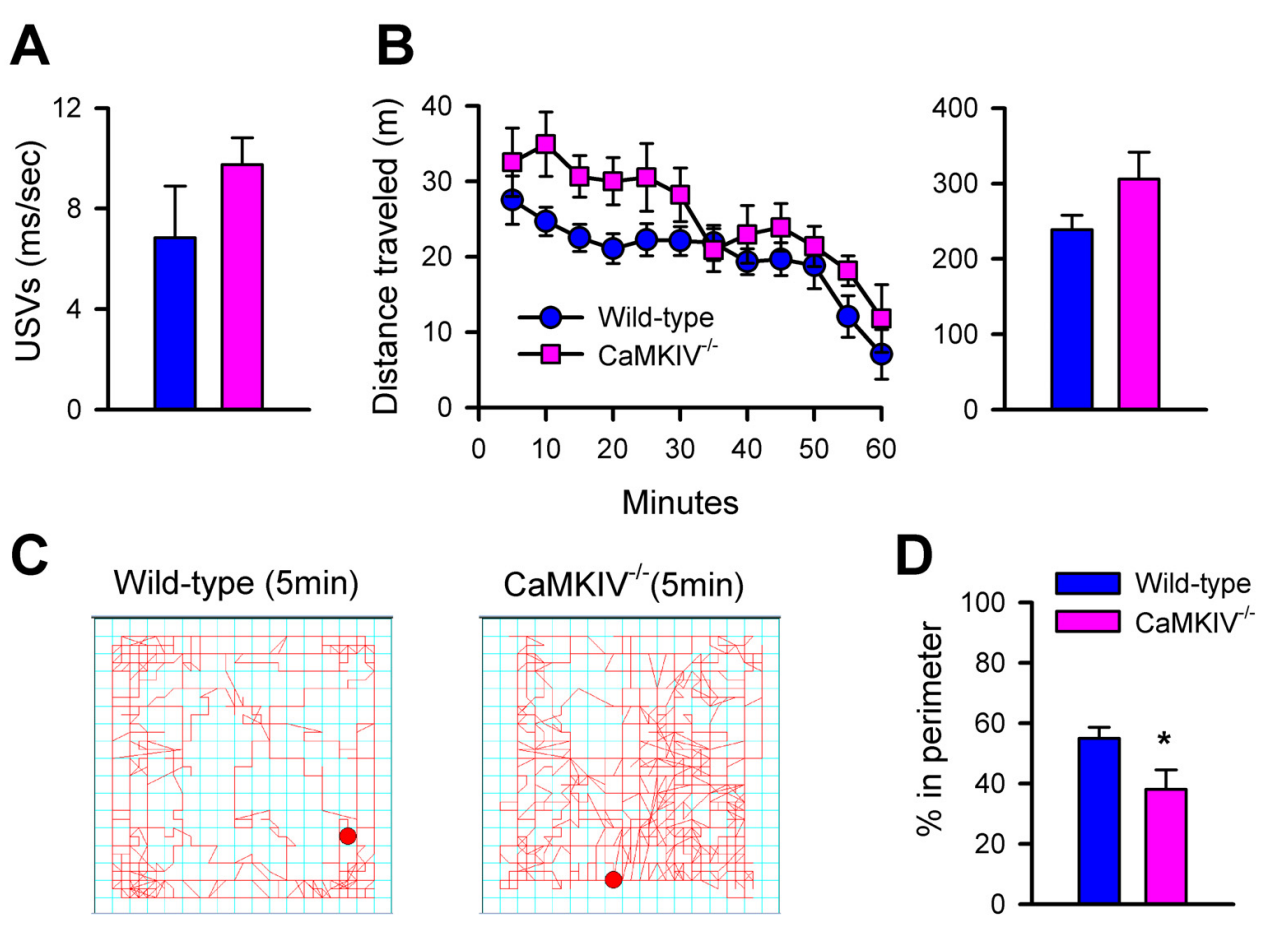
**USVs and locomotion of CaMKIV^-/- ^and wild-type mice in an open field**. **A**, 30 kHz USVs for wild-type mice (white bars, n = 11) and CaMKIV^-/- ^mice (black bars, n = 8) during 60 min in the open field. **B**, Distance traveled over 60 min in the open field represented in 5 min blocks (○, wild-type, n = 11; ■, CaMKIV^-/-^, n = 8). Right: Total distance traveled. **C**, Representative pattern of locomotion for CaMKIV^-/- ^and wild-type, illustrating the concentration of movement in the center of the open field for CaMKIV^-/- ^mice **D**, Average distance traveled in the perimeter of the open field is decreased in CaMKIV^-/- ^mice compared to wild-type mice (p < 0.05). Error bars represent S.E.M

We next measured USVs and locomotor activity in the open field for both CaMKIV^-/- ^and wild-type mice. We found no difference in 30 kHz USVs between CaMKIV^-/- ^(n = 11) and wild-type mice (n = 8) after 60 minutes in the open field (Figure 6A). Also, there were no differences in any other frequency or in the average duration of a single USV. CaMKIV^-/- ^mice did not show a difference in the total distance traveled in the open field over a one-hour period (wild-type, n = 11; CaMKIV^-/-^, n = 8; Figure 6B). This agrees with a previous study that reported no change in locomotor activity [19]. However, there was a strong concentration of movement in the center of the open field, which corresponded to a significant reduction in the percent distance traveled in the perimeter of CaMKIV^-/- ^mice (Figure 6C,D). The increased activity in the center of the open field implied a decrease in anxiety of CaMKIV^-/- ^over wild-type mice [37].

### USVs of CaMKIV^-/- ^mice induced by a noxious stimulus

**Figure 7:**
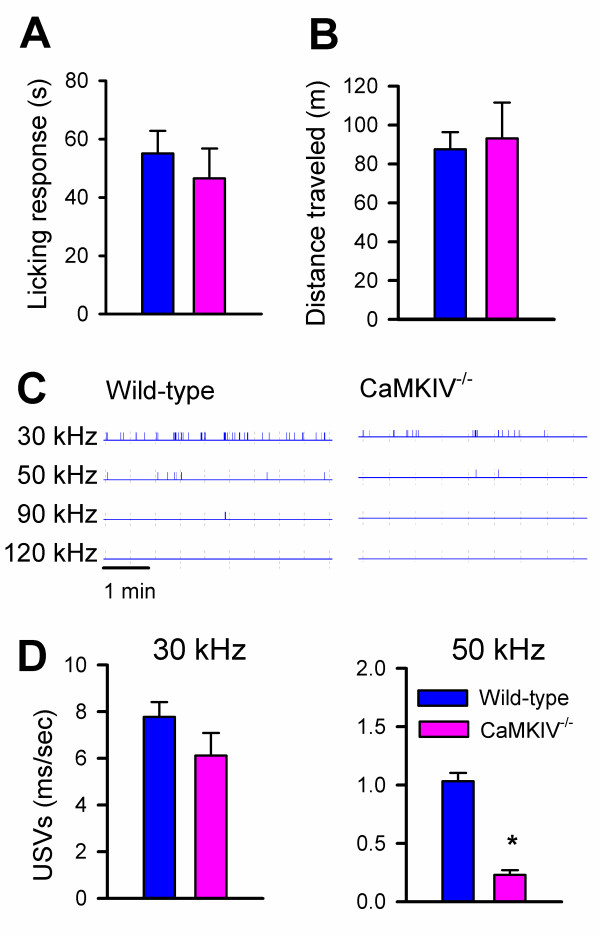
**CaMKIV is required for capsaicin-induced 50 kHz USVs but not nociceptive licking responses**. **A**, Average time spent licking the hindpaw injected with capsaicin in wild-type (white bars, n= 7) and CaMKIV^-/- ^mice (black bars, n = 6). **B**, Average distance traveled over 30 min after capsaicin injection in CaMKIV^-/- ^and wild-type mice. **C**, Representative USV trace for wild-type and CaMKIV^-/- ^mice **D**, 30 kHz and 50 kHz USVs for wild-type and CaMKIV^-/- ^mice after capsaicin injection, 50 kHz USVs are significantly decreased in the knockout receiving capsaicin (p < 0.001). Error bars represent S.E.M

Previous studies reported that CaMKIV^-/- ^mice showed no difference in their response to acute noxious stimuli. Their responses to formalin or complete Freund's adjuvant (CFA) injections, two models of inflammatory pain, were not altered compared to wild-type [10]. Here we tested the hypothesis that CaMKIV may contribute to pain-related emotional responses by examining changes in USV production in response to capsaicin injection in both wild-type and CaMKIV^-/- ^mice. We measured behavioral nociceptive responses (i.e., licking the injected hind paw), open field locomotion, and USVs after subcutaneous injection of capsaicin into the right hind paw. No significant difference in the licking responses to capsaicin was found between wild-type (n = 7) and knockout mice (n = 6, Figure 7A). There was also no difference in the total distance traveled in the open field between the two groups (Figure 7B). In wild-type mice, subcutaneous injection of capsaicin (1 μg/10 μl) into one hind paw produced a selective increase in USVs at 50 kHz (Figure 2A,B). In CaMKIV^-/- ^mice, however, the capsaicin-induced increase in 50 kHz USVs was blocked (0.58 ± 0.07 vs. 0.42 ± 0.07 ms/sec (before vs. after capsaicin)) and significantly reduced compared to 50 kHz USVs after capsaicin from wild-type mice (t = 9.25, p < 0.001, Figure 7C,D). These results suggest that the contribution of CaMKIV to pain-induced vocalization is quite selective. Behavioral withdrawal responses to acute thermal or mechanical noxious responses or inflammation do not require the activity of CaMKIV [10], but emotional responses (i.e., USVs) may require the involvement of CaMKIV.

### USVs from CaMKIV^-/- ^mice after fear conditioning

**Figure 8:**
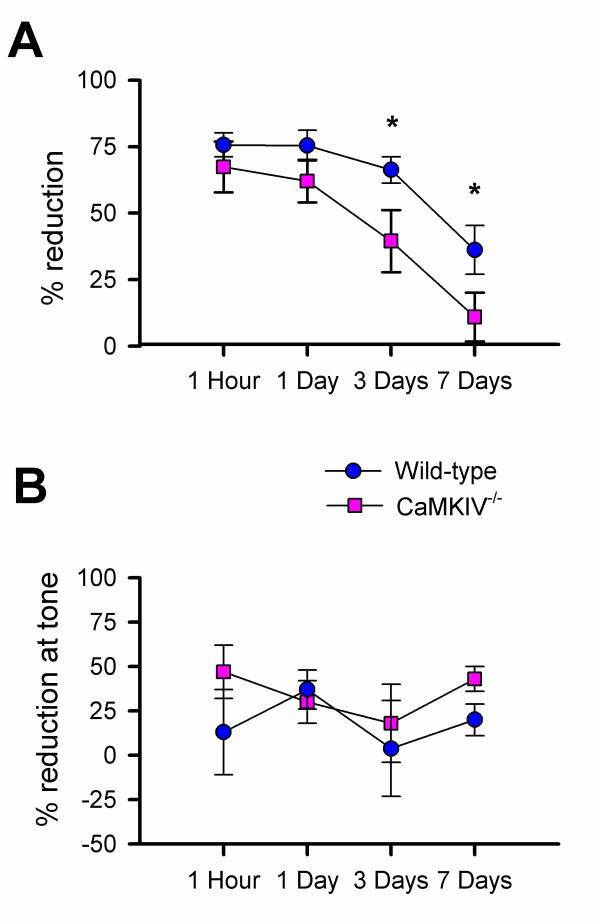
**CaMKIV contributes to the change in USVs after contextual fear conditioning**. **A**, 30 kHz USVs emitted in the contextual environment 1 h, 1, 3, and 7 days after training for wild-type and CaMKIV^-/- ^mice, expressed as the percent reduction from baseline (○, wild-type, n = 17, n = 5 for day 7; ■, CaMKIV^-/-^, n = 15, n = 5 for day7). CaMKIV^-/- ^show significantly less reduction by 3 days after training (p < 0.05). **B**, 30 kHz USVs emitted to the tone 1 h, 1, 3, and 7 days after training for wild-type and CaMKIV^-/- ^mice, expressed as the percent reduction from baseline (○, wild-type, n = 17, n = 5 for day 7; ■, CaMKIV^-/-^, n = 15, n = 5 for day7). Error bars represent S.E.M.

CaMKIV^-/- ^mice have a defect in fear memory, as measured by the behavioral freezing response [10]. To examine if CaMKIV also contributes to fear conditioning-related changes in USVs, we measured USVs from wild-type and CaMKIV^-/- ^mice during and after fear conditioning. There was no difference in USVs emitted from the knockout (n = 15) before or after the foot shock as compared with wild-type mice (n = 17). The increase in USVs during the foot shock was also unchanged in the knockout. At one hour and one day after fear conditioning, both wild-type and CaMKIV^-/- ^mice showed similar reduction in USVs when they were placed back to the same conditioning chamber. However, by three days after fear conditioning, CaMKIV^-/- ^mice showed significantly less reduction in USVs as compared with wild-type mice (t = 2.20, p < 0.05; Fig. 8A). A two-way analysis of variance (ANOVA) revealed a significant effect of genotype [F(1,101) = 8.01, p < 0.01)] and time [F(3,101) = 8.53, p < 0.001], with no interaction effect, in the context after fear conditioning. In contrast, there was no difference between CaMKIV^-/- ^and wild-type mice when USVs were measured in response to the auditory tone (Fig. 8B). While USVs were decreased at the onset of the tone, there was not a significant difference at any time point after conditioning in the knockout.

## Discussion

In the present study, we describe USVs from isolated adult male mice under different conditions. We show that the majority of USVs emitted in the open field occur within the 30 kHz range (26–34 kHz) and that the duration of a single call varies between frequencies. A noxious hind paw injection of capsaicin decreased locomotion and had no effect on 30 kHz USVs while significantly increasing 50 kHz USVs. While the foot shock significantly increased USVs at all frequencies, 30 kHz USVs made up the vast majority of all vocalization before and after fear conditioning. Additionally, 30 kHz USVs were significantly decreased in the context after conditioning and at the onset of the tone in a novel environment. CaMKIV^-/- ^mice did not differ from wild-type in vocalization in the open field or in 30 kHz USVs in response to capsaicin. However, the increase in 50 kHz USVs found in the wild-type in response to capsaicin was absent in the knockout, while licking and locomotor responses were unchanged. No difference was found in USV production during training or auditory conditioning; however, USVs were significantly less reduced in CaMKIV^-/- ^mice in the context where they had received the shock-tone pairing three days prior. Taken together, these results suggest a role for CaMKIV in the vocal behavioral responses to capsaicin and fear conditioning, and imply that the USV response can distinguish physiological responses to stimuli.

We have previously reported that CaMKIV^-/- ^mice have unaltered responses to acute noxious stimuli while having a defect in fear memory [10]. However, these results were based solely off of traditional measures of pain and fear-related behaviors (i.e. licking and freezing). Here, we employ an additional measure (USVs) to characterize the role of CaMKIV in the behavioral responses to pain and fear. A great deal of evidence supports the use of USVs as an emotional indicator in rats; they have been used as a measure of anxiety, fear memory, and as a tool to predict the impact of drug withdrawal [25,27,28]. Mice also use USVs as a form of communication, thus, it is reasonable to assume that mice would emit USVs in response to physiologically relevant events. Advances in genetic technology have increased the production of transgenic mice and USV analysis may allow for a more thorough neurobehavioral characterization. Also, current methods of behavioral observation require manual scoring and direct observation, which include human error and bias. We propose that USV analysis of mice will compliment existing methods of behavioral characterization.

In order to use USVs in mice as a useful measure of behavior, we first observed USV production at four frequency ranges in the open field. We chose to observe USVs along with open field activity since it allows us to directly correlate exploratory and vocal behavior, and provides a good context in which to observe responses in an isolated environment. Patterns of movement in the open field are widely regarded to reflect anxiety and provide a quantifiable and unbiased measure when considering the effects of various treatments on behavior. In general, there is an inverse relationship between activity and anxiety in the open field. Therefore, simultaneous recording of open field locomotor activity and vocalization will allow us to compare changes in USVs with changes in anxiety-related activity levels.

Both saline and capsaicin injections decreased locomotor activity in the open field when compared to animals that did not receive an injection (Fig 2C,D). Capsaicin has also been shown to decrease exploratory activity in rats [38]. However, only capsaicin-injected animals showed an increase in 50 kHz USVs, while other frequencies were unchanged as compared to controls and saline-injected mice (Fig. 2A,B). This suggests that the stress from handling and the hind paw injection decreases locomotor activity in mice whether they receive capsaicin or saline. The fact that only capsaicin-injected animals show an increase in 50 kHz USVs suggests that this is a response to the noxious character of the capsaicin and not to the injection itself. CaMKIV^-/- ^mice, which have previously been shown not to have a defect in motor responses to noxious stimuli [10], were similar to wild-type mice in the reduction of locomotor activity as well as in the behavioral licking response to capsaicin (Fig. 6A,B). Again, the only difference between the groups occurred at 50 kHz USVs. In this case, CaMKIV^-/- ^mice failed to show the 50 kHz increase in response to capsaicin, while USVs were unchanged at other frequencies (Figure 7C,D). These results imply that CaMKIV may play a role in emotional, and not motor, responses to noxious stimuli. Also, 50 kHz USVs may index a negative state in response to capsaicin that is independent of locomotor and licking responses. The increase in 50 kHz USVs was also seen after formalin injection (Figure 3), strengthening our hypothesis that 50 kHz USVs may reflect the animal's reaction to the noxious character of the injection.

Several studies report that 50 kHz USVs may represent a positive affective state in rats [25,35,39]. While we cannot speculate as to why there would be such a species difference in the modulation of 50 kHz USVs, we suggest that mice and rats use distinct frequencies to communicate negative and positive affective states. For example, 50 kHz USVs are emitted from male rats when mounting the female [22], in contrast, male mice emit 70 kHz USVs when mounting [29]. A previous study reports that USVs are notaffected by carrageenan induced inflammation, or chronic pain caused by Complete Freund's adjuvant (CFA), when measured in rats [40]. Further studies are needed todetermine if these models of chronic pain can elicit a change in 50 kHz USVs in mice.

Fear memory of a noxious foot shock was measured by recording behavioral freezing and was shown to be decreased in CaMKIV^-/- ^mice [10]. In these experiments, we used the same procedures, but instead measured USVs. USVs have been used along with freezing, heart rate, locomotion, and defecation as a measure of fear in rats after conditioning [41]. CaMKIV^-/- ^mice did not differ in their response to the foot shock or in vocalization to the tone as compared to wild-type. However, the knockout showed significantly less reduction in 30 kHz USVs in the context three days after fear conditioning. This suggests that changes in USVs after fear conditioning were more sensitive to the context than to the auditory tone, and that USVs are quite different from behavioral freezing responses in that no difference has been reported between contextual and auditory fear memory in CaMKIV^-/- ^mice.

We propose that 30 kHz USVs can be used as a measure of fear memory in mice. Freezing responses in CaMKIV^-/- ^mice were decreased by one day after fear conditioning [10], but USVs were not significantly decreased until three days after conditioning in this study. USVs after fear conditioning in rats are considered to be a slowly acquired measure of fear memory since three training sessions were required to condition them to the context [41]. It is possible that USVs, as a measure of fear memory in mice, may be a slower measure than the freezing response. The decrease in USVs emitted in the context after conditioning was still present seven days later, further studies are needed to determine how long this affect lasts.

In summary, our results demonstrate that capsaicin, formalin and fear conditioning induced changes in vocalization at different frequencies. In the case of an acute insult like capsaicin, enhancement of vocalization is mostly at 50 kHz. For fear conditioning, only vocalizations at 30 kHz were dramatically altered. We also report a role for CaMKIV in mediating responses to emotional stimuli. CaMKIV^-/- ^mice did not emit capsaicin-induced 50 kHz vocalizations while having the same behavioral response measured by licking. Lastly, CaMKIV^-/- ^mice showed an increase in vocalization over the wild-type three days after fear conditioning. This correlates with our previous results in CaMKIV^-/- ^mice that show a decrease in fear memory, and the fact that CaMKIV is widely distributed in cognition-related forebrain areas [10].

CaMKIV is an important member of the signal transduction pathway leading to CREB activation and gene expression and has been reported to participate in activation of the ERK pathway [42]. We believe that CaMKIV plays a role in mediating emotional responses to painful and fearful stimuli and that USV analysis will provide a model by which we can quantitatively measure an animal's internal affective state. The current manuscript focuses on characterizing USVs as a response to a noxious chemical injection and as a measure of fear memory. The evaluation of anxiolytic and analgesic drugs is important and should be considered in future studies. Our present results suggest that USV analysis may allow us to elucidate the molecular mechanisms underlying emotional responses.
